# Temporomandibular Disorders: The Habitual Chewing Side Syndrome

**DOI:** 10.1371/journal.pone.0059980

**Published:** 2013-04-08

**Authors:** Urbano Santana-Mora, José López-Cedrún, María J. Mora, Xosé L. Otero, Urbano Santana-Penín

**Affiliations:** 1 Department of Stomatology, Faculty of Medicine and Odontology, University of Santiago de Compostela, Santiago de Compostela, La Coruña, Spain; 2 Oral and Maxillofacial Surgery Service, University Hospital of La Coruña, La Coruña, Spain; 3 Department of Statistics, Faculty of Medicine and Odontology, University of Santiago de Compostela, Santiago de Compostela, La Coruña, Spain; UCLA, United States of America

## Abstract

**Background:**

Temporomandibular disorders are the most common cause of chronic orofacial pain, but, except where they occur subsequent to trauma, their cause remains unknown. This cross-sectional study assessed chewing function (habitual chewing side) and the differences of the chewing side and condylar path and lateral anterior guidance angles in participants with chronic unilateral temporomandibular disorder. This is the preliminary report of a randomized trial that aimed to test the effect of a new occlusal adjustment therapy.

**Methods:**

The masticatory function of 21 randomly selected completely dentate participants with chronic temporomandibular disorders (all but one with unilateral symptoms) was assessed by observing them eat almonds, inspecting the lateral horizontal movement of the jaw, with kinesiography, and by means of interview. The condylar path in the sagittal plane and the lateral anterior guidance angles with respect to the Frankfort horizontal plane in the frontal plane were measured on both sides in each individual.

**Results:**

Sixteen of 20 participants with unilateral symptoms chewed on the affected side; the concordance (Fisher’s exact test, P = .003) and the concordance-symmetry level (Kappa coefficient κ = 0.689; 95% confidence interval [CI], 0.38 to 0.99; P = .002) were significant. The mean condylar path angle was steeper (53.47(10.88) degrees versus 46.16(7.25) degrees; P = .001), and the mean lateral anterior guidance angle was flatter (41.63(13.35) degrees versus 48.32(9.53) degrees P = .036) on the symptomatic side.

**Discussion:**

The results of this study support the use of a new term based on etiology, “habitual chewing side syndrome”, instead of the nonspecific symptom-based “temporomandibular joint disorders”; this denomination is characterized in adults by a steeper condylar path, flatter lateral anterior guidance, and habitual chewing on the symptomatic side.

## Introduction

The cause of temporomandibular disorders is still unknown, [1] but it is considered multifactorial and includes both physical (peripheral) and psychosocial (central) factors. [2] The suggested chief etiologic factor is TMJ overloading, [3] resulting in the collapse of joint lubrication and the generation of free radicals, thereby causing hypoxia when capillary perfusion pressure is exceeded. [4] Overloading of the TMJ can originate in the masseter muscles, [5]–[7] mainly on the nonworking side, [8] and can initiate remodeling. [9]–[12].

The teeth, the main agents of food mastication, are nonrigidly articulated to the jawbones through a gonfosis (from “gonfos”, clove), which includes the periodontal ligament whose function it is to distribute the occlusal forces. [13].

Normal mastication in humans favors one side and then the other. Chewing consistently on the same side is referred to as the preferred or habitual chewing side or masticatory laterality and seems to be controlled by the central nervous system (CNS). [14], [15].

During the last phase of the chewing cycle, the jaw follows an anteromedial direction to reach the occlusal phase in which the food is chewed. A triangle supports the jaw motion. The first point of the triangle is the almost static fulcrum of the working (chewing) temporomandibular joint; and the other 2 points are the more dynamic nonworking side temporomandibular joint, [16] determining the condylar path, and the teeth, determining the lateral anterior guidance. [17].

The relationship between the use of one habitual chewing side and the dynamic peripheral factors involved in temporomandibular disorders is not fully understood. [15], [18]–[21].

In this study, we tested the null hypothesis that 1) there is no association between the symptomatic side and the side of habitual chewing and 2) that the condylar path and the lateral anterior guidance angles did not differ between the symptomatic and non-symptomatic side among individuals with chronic unilateral temporomandibular disorder.

## Methods

The Regional Human Ethics Committee of Galicia approved this study; all participants provided their written, informed consent.

### Subjects

This study investigated 507 patients who were referred to the Maxillofacial Surgery Service of La Coruña University Hospital, Spain seeking therapy for their chronic (over 6 months) pain and/or limited (up to 40 mm) jaw opening [22] and who had been diagnosed with chronic Axis I unilateral temporomandibular disorders [23] between September 2007 and July 2009.

Inclusion criteria were that the participant be aged between 18 and 65 years, be fully dentate (except for third molars and/or 4 first or second premolars for orthodontic reasons), have clinically anatomic normal occlusion, absence of dental decay, or active periodontal disease, had had no orthodontic therapy during the previous 2 years, had had no traumatic or oncologic injury in the maxillofacial area, and were fully willing and able to cooperate. Participants with other previous or current conventional therapies or different concomitant pathologies such as bruxism, fibromyalgia, or neuropathic symptoms were not excluded; 392 patients did not meet these inclusion criteria. The exclusion criterion was a requirement for extensive or traumatic selective grinding to achieve an equilibrated occlusion. A total of 115 participants who fulfilled the inclusion criteria were randomly selected and carefully evaluated (by Dr S-P); 94 participants were excluded due to their occlusal condition. No patients declined to participate. Twenty-one participants (mean age 32.70(11.29) years, range 19–58 years; 17 women and 4 men) were included in this study. One female presenting with bilateral symptoms was excluded from between-sided comparisons to simplify the discussion.

### Chief Complaint

Pain intensity during the previous weeks, including the examination day, was marked by each patient on a 0 to 10 cm graded visual analog scale. [1], [24].

Maximum unassisted mouth opening between the maxillary and mandibular incisors was measured with a Boley gauge. [22] Before the study began, the interobserver reliability of the 2 investigators was tested by comparing their measurements of a set of 25 subjects; this yielded an intraclass correlation coefficient (ICC) of 0.95.

### Chewing Assessment

Four tests were conducted in each individual: 1) intraoral exploration of the side with more pairs in contact during lateral jaw motion, [25] which is usually toward more horizontal motion; 2) direct inspection while eating toasted almonds; 3) with kinesiography (K6-I Diagnostic System, Myotronics-Noromed, Inc., Kent, WA) of the masticatory movement with chewing gum [26]; and 4) by means of interview [27] to assess both current and previous chewing patterns. Participants with consistent alternate chewing or contradictory results on some tests were considered as alternates for the study.

### Axiography

**Figure 1:**
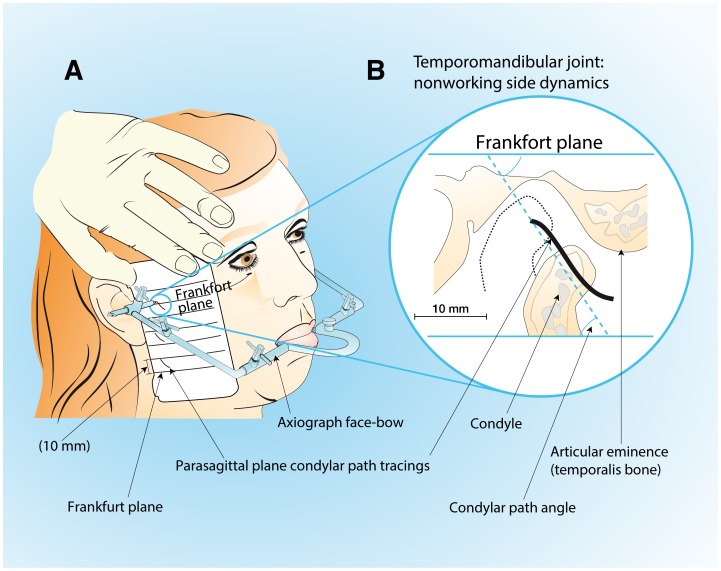
Axiography procedure: condylar path tracings. A, the kinematic face-bow attached with silicone putty to mandibular teeth through an occlusal rim; lateral condylar path drawn on the surface of the recording card. B, parasagittal plane of lateral condylar path tracings and their angle with respect to the tragus-infraorbital Frankfort plane.

Condylar path tracings in relation to the Frankfort line were made in the parasagittal plane by following the Gysi extraoral method. [28] A kinematic Gerber’s face-bow (Kit Registier Ausrustung “C”; Condylator service, Zurich, Switzerland) was used [29] (Fig. 1); measure repeatability was excellent (ICC = 0.92).

### Kinesiography

**Figure 2:**
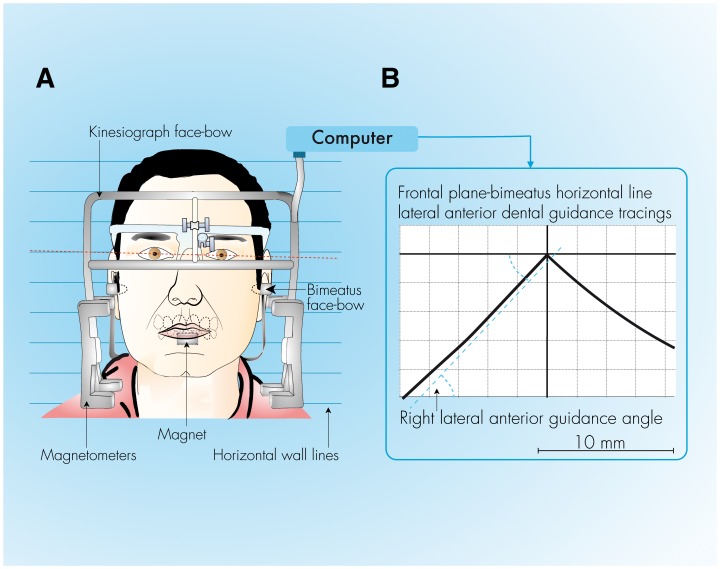
Gnathography procedure. A, The face-bow placed on the patient’s head and the magnet attached to the buccal surface of the mandibular incisors. B, lateral anterior dental guidance tracings and the right angle with respect to the *bimeatus*-horizontal plane.

Lateral jaw motion in the frontal plane was recorded with a calibrated Model K6I diagnostic system (Fig. 2). The angle between the tangent of the lateral anterior guidance tracings and the horizontal (bimeatus) Frankfort line, starting from the midsagittal point up 2 mm, [17] was measured with a goniometer-protractor. The interobserver repeatability of the measurements was closely repeatable (ICC = 0.96). Two different examiners assisted in performing each series of 3 tests. The tests were conducted in a double-blind manner; participants were unaware of the objectives of the assessment; and the clinicians were unaware of the participant’s condition.

### Statistical Analysis

Values of continuous variables are presented as means (standard deviation). The associations between the side of habitual chewing and the side of chronic symptoms was analyzed with Fisher’s exact test and the concordance symmetry-level was assessed with the “κ” Kappa coefficient. Axiographic and kinesiographic results were compared by the unpaired (interindividual) or the paired (intra-individual**)** two-tailed Student *t*-test. The alpha level was set at.05.

With a sample size of 19, and assuming a two-sided test at the 0.05 level, the study had power of 0.8 to detect a value of Kappa of 0.6 or larger when testing the null hypothesis that Kappa = 0. [30].

## Results

### Chief Complaint

Twelve participants suffered chronic symptoms on the right, 8 on the left, and 1 on both sides; this last participant was excluded from intra-individual comparisons. Mean self-reported pain intensity was 5.36(2.42), (range 0–9). The pain was continuous in 14 patients (70%); however, 1 female participant experienced chronic pain only during maximum mouth opening (limited to 31 mm).

The mean maximum spontaneous mouth opening was 39.42(8.92) mm, (range 24–65 mm). Limited mouth opening (≤40 mm) was present in 14 of the 21 (65%) participants. The mean maximum unassisted forced mouth opening was 42.60(8.94) mm, (range 29–65) and limited in 9 (43%) participants. There were no gender or involved-side differences.

### Habitual Chewing and Pain-sided Correlations

Participants were grouped into right-sided (n = 8), left-sided (n = 12), and alternate-sided (n = 1) chewers. There was a significant association between the habitual chewing side and the pain side (Fisher’s exact test, P = .003); the Kappa coefficient showed significant concordance between the side habitually used to chew and the side with chronic symptoms (κ = 0.69; 95% CI, 0.38 to 0.99; P = .002).

### Axiography

The mean condylar path angle was 49.41(10.04) degrees, range 34 to 75 degrees, with no differences between sides. Condylar path angles were significantly steeper on the symptomatic side, 53.47(10.88) degrees versus 46.16(7.25) degrees, P = .001; and on the habitual chewing side, 50.78(9.47) degrees versus 45.61(7.29) degrees, P = .005.

### Kinesiography

The mean lateral anterior guidance angle of the frontal plane was 45.05(11.78) degrees, range 12 to 72 degrees, with no differences between sides. The mean lateral anterior guidance angle was significantly flatter on the pain side, 41.63(13.35) degrees versus 48.32(9.53) degrees, P = .036 and on the habitual chewing-side 41.56(12.16) degrees versus 49.61(10.61) degrees; P = .014.

## Discussion

This is the first study to show that chronic unilateral temporomandibular disorders mainly affect the habitual chewing side, the side which also exhibits a higher condylar path and flatter lateral anterior guidance angles.

**Figure 3:**
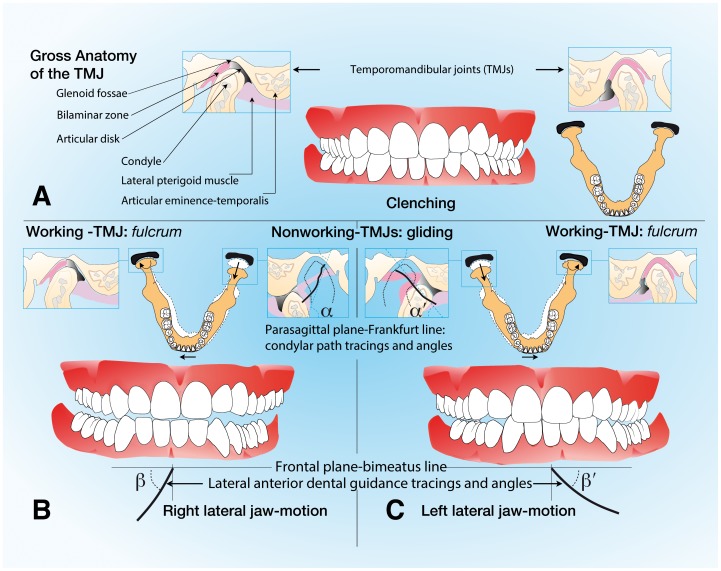
Craniomandibular relationships of a patient with left-side symptoms. A, Maximal intercuspal position. B, Right lateral jaw motion. C, Left lateral jaw motion. Left lateral jaw motion is more horizontal than right lateral jaw motion (α>α’ and/or β>β’).

This statistical correlation allows a hypothesis that the habitual chewing side could be a contributing factor to temporomandibular disorders, and, according to other studies, [11], [12] that this leads to specific remodeling of the chewing apparatus. Consequently, it is plausible to assume a specific diagnosis, the habitual chewing side syndrome, which is characterized by habitual chewing, a steeper condylar path, and flatter lateral anterior guidance on the affected side (Fig. 3).

### Participants

Because of the controversy concerning the influence of peripheral dental factors on temporomandibular disorders [18] and on the habitual chewing side, [14], [15] only completely dentate participants with normal occlusion and suffering from chronic temporomandibular disorders were randomly selected. [23] This sample was a subset of participants under the care of a public hospital with a catchment area of more than one million people and so can be considered representative of the general population.

### Chewing

There is no validated test to assess chewing function. Clinical tests generally fail to establish chewing function retrospectively. The interview, [27] which attempted to elicit information about unconscious function, was sometimes unsuccessful. Because the chewing function seems to influence the alteration, remodeling, and development of the stomatognathic structures, [11], [12] in this study, a particular effort was made to analyze it carefully by implementing several tests.

The association between habitual chewing side and temporomandibular disorders has been reported previously. [19], [20] Unfortunately, in those studies, the authors did not report whether the side used to chew was associated with the affected side; moreover, the first of these studies included participants with missing teeth, and the second included participants with occlusal pathology. Despite these methodological differences, the present study seems to agree with those previous findings and confirmed that the participants habitually chewed on the affected side.

Unilateral chewing implies asymmetry of joint dynamics [16] and load distribution. [7] The joint that performs the more extensive motion (nonworking side) is lubricated and can exchange metabolites better than the side that does not move (working side); however, only the teeth on the working side are stimulated, [13] so the stomatognathic structures can only benefit if both sides alternate in performing the chewing function.

Using one habitual chewing side is common in the general population. [21] This could explain, on the one hand, the presence of damage to the TMJ in asymptomatic individuals, [31] and on the other hand, the impossibility of establishing a cut-off to identify healthy patients who are likely to be affected because of the multiplicity of causal factors and fluctuating nature of the symptoms. These seem to depend on the biomechanics of the masticatory dysfunction specific to each individual and/or psychobiological conditions. [2] The habitual chewing side appears to be associated with temporomandibular disorders but may not be sufficient per se to cause symptoms.

### Axiography

The mechanical device used in this study is inexpensive, does not require previous casts and clutches, is not time consuming, is methodologically reproducible, [29] and can be easily applied in the clinical setting.

This is the first study showing intra-individual condylar path side dimorphism in those with chronic unilateral temporomandibular disorders. Condylar path asymmetry is probably an adaptive mechanism caused by the predominant use of one side. [12] It is hypothesized that the increased condylar path (increasing eminence) causes difficulty and limits the motion of the condyle needed on the nonworking side, [16] which helps perpetuate the choice of the habitual chewing side. Moreover, since remodeling of the TMJ occurs slowly in response to biomechanical demands, the habitual chewing side is probably an associated factor rather than a consequence; though, of course, some patients avoid using one side because of pain (2 instances in this study).

### Kinesiography

The diagnostic value of the lateral anterior guidance angles should be interpreted cautiously because some lateral jaw movements are pathologically guided by the opposite side (nonworking side interference), and in any given individual, the anterior dental anatomy may be modified because of oral rehabilitation, orthodontics, or tooth loosening).

The side exhibiting the temporomandibular disorder also shows a flatter lateral anterior guidance angle. A clinical association was previously demonstrated between a flatter lateral anterior guidance angle and temporomandibular disorders in asymptomatic patients, suggesting that flat lateral anterior guidance angles do not sufficiently protect the ipsilateral TMJ. [17] This study provides the basis of a different explanation for this association in a different way by suggesting that the symptoms are a consequence of the biodynamics resulting from the use of one habitual chewing side. Moreover, the higher range values and SD in the present study suggest higher intra-individual variability in lateral anterior guidance angles (range 34 to 72 degrees with intra-individual differences reaching up to 25 degrees), which, in turn, suggests severe masticatory dysfunction in chronic symptomatic unilateral TMD patients.

### Pathophysiological and Etiopathogenetic Considerations

There are 2 distinct features of the habitual chewing side syndrome: increased masseter activity and reduced TMJ motion. Because the masseters are responsible for TMJ loading, [5] mainly on the nonworking side, [7] the TMJ of the habitual chewing side could be overloaded when acting as the nonworking side (when the patient uses the non-habitual chewing side); moreover, the chronic reduction in condylar motion could suddenly change and perform a larger trajectory. [16] These alterations in biomechanics could lead to overloading of the TMJ and consequent internal damage and/or pain.

This study does not support the dominant effect of the CNS on the choice of the habitual chewing side. [15] On the contrary, the present results seem to confirm Hildebrand’s assertion that the subject chooses the side where most teeth are in contact during lateral gliding [25] and where the lateral anterior guide is more horizontal and strongly suggest the influence of peripheral factors. Although the CNS influence does exist and does not change throughout life, it is likely that the CNS possesses the organization and plasticity to “decide” to chew on the side of the mouth that is better prepared or perhaps less uncomfortable.

The flattening of the chewing side anterior guidance angle could be a consequence and/or cause of the habitual chewing side. However, condylar path remodeling can only appear after a long period of altered chewing function; thus, it can only be a consequence.

### Limitations of this Study

Clinical tests evaluate masticatory function in an artificial environment and at only one time. The feedback from the interview aimed to minimize this problem.

This study was based on diagnostic reports of patients involved in one randomized trial designed to test the therapeutic efficacy of occlusal adjustment and does not include a healthy control group. Neither the habitual chewing side nor the lateral anterior guidance or condylar path angles allow us to discriminate “healthy” patients from those with temporomandibular disorders (lack of specificity/sensitivity). However, they usually allow differentiation of the symptomatic side in a given patient.

Because this study is cross-sectional, it does not permit elucidation of whether the condylar path increases in steepness on the chewing-side or whether the flattening on the nonchewing side predominates. Genetic condition, [2] physiological and pathologic remodeling or degenerative changes of the bone, [10] the influence of the masticatory function, [11], [12] and the biodynamics of one habitual chewing side all interact in an intricate but as yet unelucidated way.

The restrictive eligibility criteria were an attempt to standardize the sample to avoid potential confounders. This led to the need to screen a larger sample. and the results probably demonstrate more internal validity; however, the external validity was not affected because all inclusion and exclusion criteria were established before the outcomes assessment.

These results can only be partially extrapolated to individuals with compromised dental status.

### Clinical Implications

The diagnosis of temporomandibular disorders should probably include a determination of alternate versus one particular habitual chewing side. Healthcare givers should be aware that this condition, which could go unnoticed by the patient. This study suggests that it is not enough to maintain anatomic tooth health, but that other dynamic and functional factors should be taken into account to avoid temporomandibular disorders.

From the diagnostic point of view, independent of dental status, condylar path asymmetry suggests habitual chewing on the steeper side, although this can be altered by pathological conditions. From a therapeutic point of view, rehabilitation should, ideally, facilitate/prioritize the chewing function on the previously non- chewing and unaffected side to improve the TMJ/muscle dynamics and lead to subsequent remodeling.

*Regarding lateral anterior guidance,* from the diagnostic point of view, habitual chewing function usually occurs on the more horizontal side, but the clinician should investigate whether lateral guidance angles had been recently altered. From the therapeutic point of view, we expect that reducing the steeper lateral guidance angle (sometimes increasing the flatter one) will reestablish physiologic mastication. Moreover, more factors determining the habitual chewing side exist, for example, hemispheric dominance. The therapeutic alteration of lateral guidance should be performed carefully and sequentially to fin the minimum reduction necessary to improve chewing. Also, any therapy designed to achieve physiologic mastication should be performed after physiologic jaw closure is established.

These issues deserve further investigation.

It is hypothesized that the impaired masticatory biodynamic, TMJ remodeling, and lateral anterior guidance asymmetry develop slowly, and the symptoms usually appear only after some time; therefore, the symptoms can only be a consequence rather than a cause of the peripheral factor asymmetries.

The nonspecific label of temporomandibular disorders should be re-examined, and a new etiological entity, the habitual chewing side syndrome, should be applied.

In conclusion, this study strongly suggests that unilateral chronic temporomandibular disorders affect the habitual chewing side, which is the side with a steeper condylar path and flatter lateral anterior dental guidance.

It is often stated that “associations never prove a causality,” and the true proof of any theory requires substantial experimental data. Lateral jaw motion could be experimentally altered, with the expectation of improving the chewing function and relieving the symptoms of chronic temporomandibular disorders. This will be addressed in a randomized clinical trial.

### Search Strategy and Selection Criteria

The Cochrane database systematic reviews and the MEDLINE database were searched by using PubMed to identify articles containing “temporomandibular disorders”, “temporomandibular joint disorders”, “myofascial pain”, “arthralgia”, “osteoartrosis”, “degenerative joint disease” “orofacial pain”, “disc derangement”, “condylar path”, “anterior guidance”, “preferred chewing side”, “dental occlusion”, “etiology”, “risk”, “therapy”, “reviews”, “randomized clinical trial” up to July 2012. As additional sources and crosschecks for the reliability of the search strategy, we reviewed comprehensive textbooks on orofacial pain, temporomandibular disorders, prosthodontics, and occlusion.
